# Efficacy of atomoxetine versus midodrine for neurogenic orthostatic hypotension

**DOI:** 10.1002/acn3.50968

**Published:** 2019-12-19

**Authors:** Jung‐Ick Byun, Do‐Yong Kim, Jangsup Moon, Hye-Rim Shin, Jun‐Sang Sunwoo, Woo‐Jin Lee, Han‐Sang Lee, Kyung‐Il Park, Soon‐Tae Lee, Keun‐Hwa Jung, Ki‐Young Jung, Manho Kim, Sang Kun Lee, Kon Chu

**Affiliations:** ^1^ Laboratory for Neurotherapeutics Department of Neurology Comprehensive Epilepsy Center Center for Medical Innovations Biomedical Research Institute Seoul National University Hospital Seoul Republic of Korea; ^2^ Department of Neurology Kyung Hee University Hospital at Gangdong Seoul Republic of Korea; ^3^ Program in Neuroscience Seoul National University College of Medicine Seoul Republic of Korea; ^4^ Rare Disease Center Seoul National University Hospital Seoul Republic of Korea; ^5^ Department of Neurosurgery Seoul National University Hospital Seoul Republic of Korea; ^6^ Department of Neurology Seoul National University Hospital Healthcare System Gangnam Center Seoul Republic of Korea

## Abstract

**Objective:**

The efficacy and safety of 1‐month atomoxetine and midodrine therapies were compared. Three‐month atomoxetine and combination therapies were investigated for additional benefits.

**Methods:**

This prospective open‐label randomized trial included 50 patients with symptomatic neurogenic orthostatic hypotension (nOH). The patients received either atomoxetine 18 mg daily or midodrine 5 mg twice daily and were evaluated 1 and 3 months later. Those who still met the criteria for nOH at 1 month received both midodrine and atomoxetine for an additional 2 months, and if not, they continued their initial medication. The primary outcome was an improvement in orthostatic blood pressure (BP) drop (maximum BP change from supine to 3 min after standing) at 1 month. The secondary endpoints were symptom scores, percentage of patients with nOH at 1 and 3 months.

**Results:**

Patients with midodrine or atomoxetine treatment showed comparative improvement in the orthostatic BP drop, and overall only 26.2% of the patients had nOH at 1 month, which was similar between the treatment groups. Only atomoxetine resulted in significant symptomatic improvements at 1 month. For those without nOH at 1 month, there was additional symptomatic improvement at 3 months with their initial medication. For those with nOH at 1 month, the combination treatment resulted in no additional improvement. Mild‐to‐moderate adverse events were reported by 11.6% of the patients.

**Interpretation:**

One‐month atomoxetine treatment was effective and safe in nOH patients. Atomoxetine improved orthostatic BP changes as much as midodrine and was better in terms of ameliorating nOH symptoms.

## Introduction

Orthostatic hypotension (OH) can lead to lightheadedness, weakness, dizziness, and syncope^1^, ^2^ and is associated with an increased risk of depression.^3^ Nonpharmacological treatments, including intermittent water bolus and physical countermaneuvers, may alleviate OH‐related symptoms but are not sufficient when used alone.^4^ Pharmacological treatment is often essential in managing neurogenic OH (nOH).^5^ Currently, only midodrine and droxidopa are approved by the US Food and Drug Administration (FDA) for treating nOH^6^, ^7^; however, their use is often limited by side effects or nonresponsiveness. Previously, we demonstrated that pyridostigmine can be effective for long‐term treatment of nOH; however, its efficacy was lower than that of midodrine.^8^ Alternative medications for nOH are crucial in clinical practice.

Atomoxetine is a norepinephrine transporter blocker that increases the norepinephrine concentration in the synaptic gap. It is approved by the FDA for managing attention deficit hyperactivity disorder (ADHD),^9^ and its long‐term use is well tolerated in patients with ADHD.^10^ A few studies have reported that low‐dose atomoxetine (18 mg) has an acute benefit in managing orthostatic blood pressure (BP) changes and the related symptoms in patients with nOH.^11^, ^12^, ^13^ However, the efficacy of sustained atomoxetine treatment with regard to ameliorating orthostatic BP drops and the associated symptoms has not been evaluated in detail. Moreover, whether there can be an additional benefits obtained by combining atomoxetine with midodrine has not been demonstrated.

In this study, we performed a randomized open‐label clinical trial to evaluate the efficacy and safety of atomoxetine versus midodrine for patients with nOH. First, we performed a head‐to‐head comparison of the 1‐month efficacy and safety of the two drugs. Furthermore, we evaluated whether sustained treatment for up to 3 months or treatment with a combination of the two medications would have an additional benefit.

## Methods

### Study participants

Patients 18 years or older with symptoms of orthostatic intolerance (e.g., dizziness, lightheadedness, and feeling faint) who visited the Department of Neurology of Seoul National University Hospital or Kyung Hee University Hospital at Gangdong were considered for inclusion. The inclusion criterion was symptomatic nOH determined by medical history and clinical examination. OH was defined as a systolic blood pressure (SBP) reduction of 20 mmHg or more or a diastolic blood pressure (DBP) reduction of 10 mmHg or more within 3 min of standing.^14^ The exclusion criteria were (1) OH caused by medication, such as diuretics or beta‐blockers and (2) significant systemic illness. Patients with a typical history of prodromes and triggers of vasovagal syncope were also excluded after clinical interviews by neurology experts (K.C, J.‐I.B).

This study was approved by the Institutional Review Boards (IRBs) of Seoul National University Hospital and Kyung Hee University Hospital at Gangdong (IRB No. 1409‐066‐609 and 2017‐10‐014, respectively) and was registered at http://ClinicalTrials.gov (NCT03350659). All patients provided written informed consent to participate before enrollment.

### Study design and procedures

This was a randomized open‐label parallel study. At the baseline, we obtained medical histories, performed physical examinations, and administered self‐reported questionnaires. Orthostatic BP and HR were measured as in a previous study.^8^ In short, BP was measured with a Welch Allyn BP monitor (Welch Allyn Protocol Inc., Beaverton, OR) after 10 min of rest and after standing for 1 and 3 min. Supine SBP and DBP were recorded, and maximum decrements in SBP and DBP at 3 min were calculated. The patients who met the inclusion criteria were then randomized to receive (1) atomoxetine 18 mg once a day or (2) midodrine 5 mg twice a day. Randomization was done at the Seoul National University Hospital Clinical Research Unit with a list of computer‐generated random numbers (block of size 2). The patients were reevaluated at 1 and 3 months after the treatment was initiated. If the patients still met the criteria for OH at 1 month, both midodrine and atomoxetine were prescribed for an additional 2 months, and if not, they continued their initial medication. The patients also received education regarding nonpharmacological measures to treat OH (e.g., increased water intake, high‐salt diet, isometric exercises, and other measures). The orthostatic BP and HR measurements and questionnaires were repeated, and drug compliance, possible side effects and concomitant medications were checked at each visit.

### Questionnaires

Self‐reported questionnaires, including the OH questionnaire (OHQ)^15^ and the Beck Depression Inventory‐II (BDI‐II),^16^ were administered at the baseline and at 1 and 3 months after the treatment, as in a previous study.^8^ The OHQ evaluates OH‐associated symptoms and disability. This questionnaire reflects the severity of OH‐related symptoms on a 10‐point scale, with 0 indicating the absence of a symptom and 10 indicating maximal severity, and it has two components: the OH daily activity scale (OHDAS), which contains four items measuring the impact of OH on daily activities, and the OH symptom assessment (OHSA), which contains six items measuring the symptoms of OH.^15^ The BDI‐II, which comprises 21 multiple‐choice questions, was used to evaluate depression.^16^

### Outcomes

The primary endpoint was improvement in the orthostatic BP drop at 1 and 3 months after the initiation of treatment. The maximum decrements in SBP and DBP within 3 min of standing were analyzed. The secondary endpoints were (1) the amelioration of the questionnaire score evaluating OH‐associated symptoms and depression at 1 month; (2) the percentage of patients fulfilling the OH criteria at 1 and 3 months; and (3) any additional improvement in orthostatic BP drop, OH‐associated symptoms and depression at 3 months in those who received single and combination treatments.

The safety endpoints were adverse events. Expected adverse reactions were listed in the protocol, and causality was determined by the treating physician. Adverse events were defined as any unintended response thought to be related to treatment, and the Common Terminology Criteria for Adverse Events (CTCAE v 4.0) was used to grade events. Severe adverse events were defined as those that were grade 3 or more. We also investigated proportion of patients with supine hypertension (defined by supine SBP >150 mmHg or supine DBP >90 mmHg^17^) during follow‐up.

### Statistical analysis

We used data from our previous study of midodrine in nOH patients^8^ to guide our decision regarding sample size of each groups using PASS 16 (Power Analysis and Sample Size Software, Kaysville, UT). Based on a two‐sided significance level of 0.05, a sample size of 17 data pairs achieves 80.1% power to reject the null hypothesis of zero effect size in orthostatic BP changes at 1 month of treatment when the population effect size is 0.73 and the significance level (alpha) is 0.05 using a two‐sided paired *t*‐test. To compensate for an anticipated approximate 20% withdrawal rate, we enrolled 25 participants per group.

All data are presented as the means ± standard deviation (SD). All analyses were performed on the intention to treat principle, and missing values were excluded from the analysis. Continuous data were tested for normality of distribution with the Kolmogorov–Smirnov test and are presented as the means ± SD. Continuous data were compared using t‐tests or Mann–Whitney *U* tests, as appropriate, and the chi‐square test was used to analyze categorical data. Initially, we compared differences between the atomoxetine and midodrine groups with respect to supine and orthostatic BP, HR, and questionnaire scores. Then, we evaluated changes from the baseline to 1 month by performing a Wilcoxon signed‐rank test for each treatment group. Repeated‐measures ANOVA with the group (atomoxetine and midodrine) as the between‐subject factor and time (baseline and 1 month after treatment) as the within‐subject factor was used to test for an overall difference in the treatment effects. Long‐term additional changes from 1 to 3 months were evaluated by performing the Wilcoxon signed‐rank test for each group (atomoxetine single, midodrine single, and combination group). Data were analyzed with SPSS 22.0 for Windows (SPSS Inc., Chicago, IL, USA), and the significance was set at *P* < 0.05.

## Results

### Clinical features and baseline characteristics

**Figure 1:**
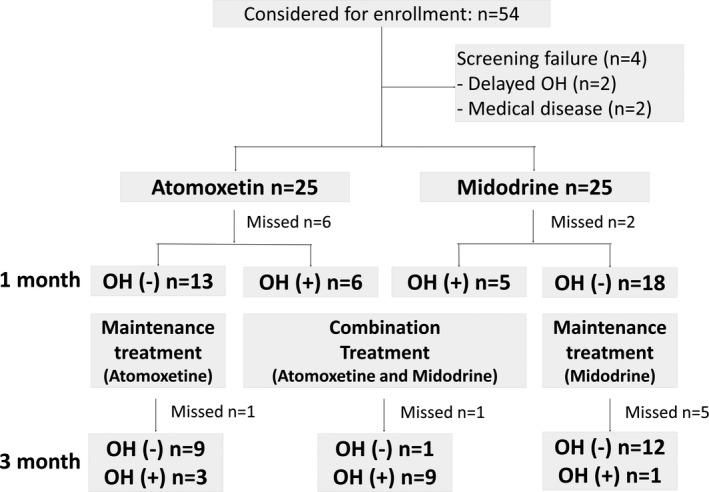
Number of patients with OH at 1 and 3 months after the administration of the study drug. Missed, missed visit; OH, orthostatic hypotension.

A total of 50 patients were enrolled and randomized into two groups (Fig. 1). The mean age was 63 years, and 28 (56.0%) were men. Five patients (10%) had nondiabetic autonomic neuropathy, 8 patients (16%) had diabetic autonomic neuropathy, 8 patients (16%) had multiple system atrophy, and 29 patients (58%) had unspecified OH. The patients were well matched by age, sex, and etiology (Table 1).

**Table 1:** Patient characteristics.

	Total	Atomoxetine	Midodrine	*P* value
	50	25	25	
Age (years)	63.1 ± 9.6	64.4 ± 7.0	61.8 ± 11.7	0.344
Sex (male)	28 (56.0)	14 (56.0)	14 (56.0)	1
Height (cm)	165.0 ± 9.4	165.9 ± 8.1	164.1 ± 10.7	0.514
Weight (kg)	67.5 ± 11.5	67.7 ± 11.3	67.2 ± 12.0	0.876
BMI (kg/m^2^)	23.7 ± 5.8	23.6 ± 6.0	23.9 ± 5.7	0.876
Etiology				0.286
Idiopathic nOH	29 (58.0)	16 (64.0)	13 (52.0)	
MSA	8 (16.0)	3 (12.0)	5 (20.0)	
Diabetic AN	8 (16.0)	4 (16.0)	4 (16.0)	
Nondiabetic AN	5 (10.0)	2 (8.0)	3 (12.0)	

Data are presented as the mean ± SD or number (percentage). BMI, body mass index; nOH, neurogenic orthostatic hypotension; MSA, multiple system atrophy; AN, autonomic neuropathy.

At the baseline, the supine BP and HR were similar between the two groups. All patients exhibited substantial decreases in SBP (mean 26.3 ± 11.1 mmHg) and DBP (mean 13.5 ± 8.5 mmHg) when changing from the supine to the upright position without substantial increases in HR (mean 11.8 ± 9.7/min). The baseline questionnaire scores were also comparable between the groups.

### Orthostatic BP drops improved in both the atomoxetine and midodrine groups at the 1‐month follow‐up visit

**Figure 2:**
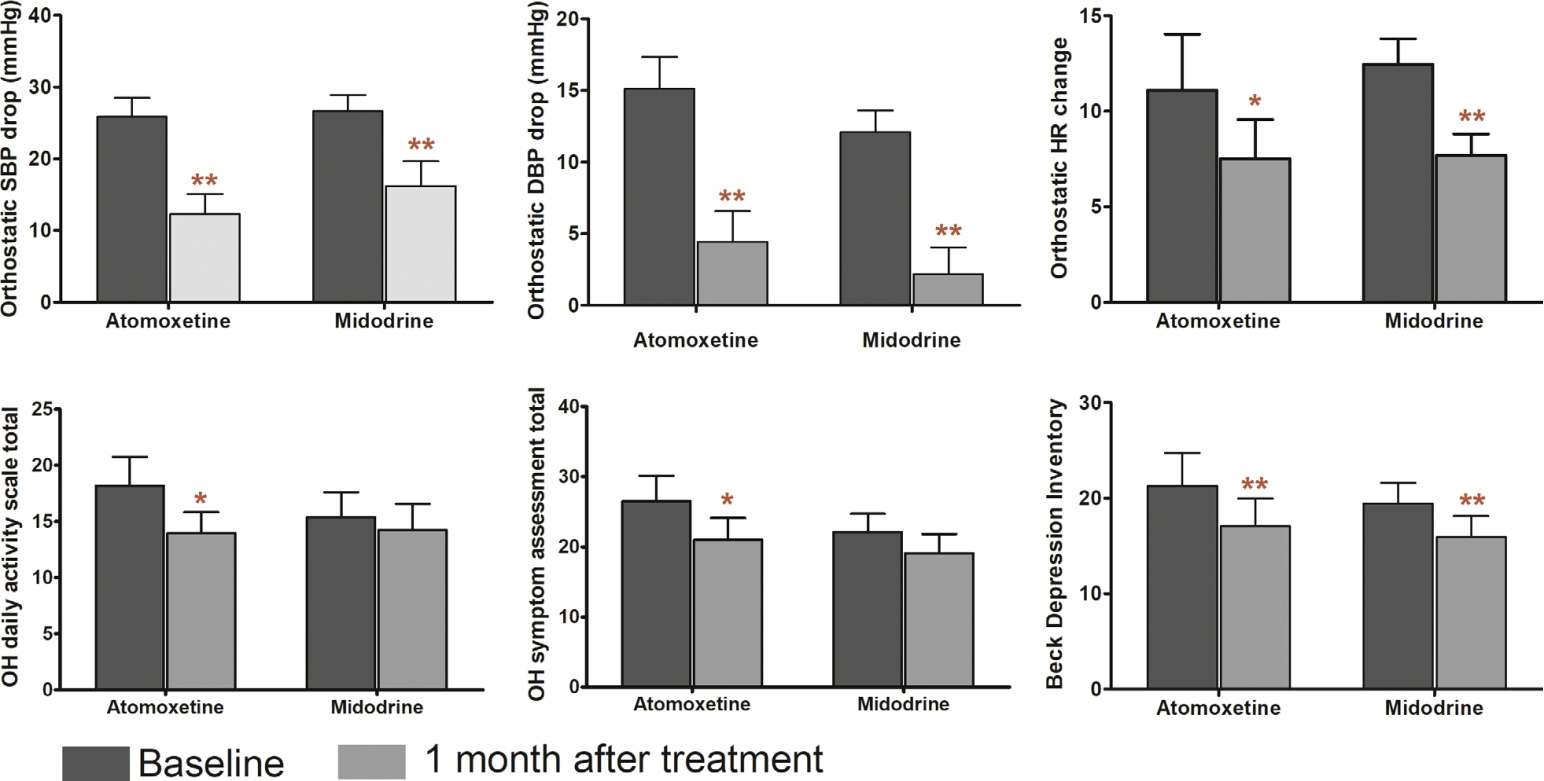
Orthostatic BP drop and questionnaire scores at the baseline and 1 month after the administration of the study drug. *P < 0.05 compared with the baseline, **P < 0.01 compared with the baseline according to the paired *t*‐test. BP, blood pressure.

After 1 month, the orthostatic SBP and DBP drops improved in each group compared to the baseline. There were no significant changes in supine SBP and DBP in either group. The supine HR significantly increased only in the midodrine group compared to the baseline (Fig. 2, Table 2).

**Table 2:** Orthostatic vital signs and Questionnaire scores at baseline and 1 month.

	Total	Atomoxetine	Midodrine	*P* value^a^
No. of patients with nOH				
Baseline	50	25	25	
1 month	11/42 (26.2)	6/19 (31.6)	5/23 (21.7)	0.470
Orthostatic SBP drop (mmHg)				
Baseline	26.3 ± 11.1	25.8 ± 11.7	26.7 ± 10.9	0.761
1 month	14.4 ± 14.9^†^	12.3 ± 12.5^†^	16.2 ± 16.7^†^	0.404
Orthostatic DBP drop (mmHg)				
Baseline	13.5 ± 8.5	15.1 ± 9.8	12.1 ± 7.3	0.418
1 month	3.2 ± 9.1^†^	4.4 ± 9.4^†^	2.2 ± 8.9^†^	0.418
Orthostatic HR increase				
Baseline	11.8 ± 9.7	11.1 ± 12.8	12.4 ± 6.5	0.849
1 month	7.6 ± 7.0^†^	7.5 ± 8.8*	7.4 ± 5.3^†^	0.425
Supine SBP (mmHg)				
Baseline	128.7 ± 18.1	126.4 ± 17.7	130.5 ± 18.7	0.940
1 month	130.4 ± 18.8	128.1 ± 17.4	132.4 ± 20.1	0.830
Supine DBP (mmHg)				
Baseline	80.3 ± 11.2	80.5 ± 12.4	80.0 ± 10.5	0.790
1 month	80.1 ± 11.5	80.5 ± 12.2	79.8 ± 11.2	0.830
Supine HR				
Baseline	68.4 ± 13.1	72.2 ± 16.8	65.3 ± 8.2	0.235
1 month	77.4 ± 13.4^†^	80.5 ± 15.2	74.9 ± 11.4^†^	0.202
OHQ total				
Baseline	40.8 ± 22.6	44.7 ± 23.9	37.5 ± 33.4	0.314
1 month	34.1 ± 21.7^†^	35.0 ± 20.1^†^	33.4 ± 23.4	0.813
OHDAS				
Baseline	16.7 ± 10.9	18.2 ± 11.2	15.4 ± 10.7	0.324
1 month	14.1 ± 9.8	14.0 ± 8.3*	14.3 ± 11.1	0.751
OHSA				
Baseline	24.1 ± 14.0	26.5 ± 15.7	22.1 ± 12.4	0.390
1 month	20.0 ± 13.3*	21.0 ± 13.6*	19.1 ± 13.2	0.640
BDI				
Baseline	20.3 ± 12.6	21.3 ± 15.1	19.4 ± 10.3	0.733
1 month	16.4 ± 11.4^†^	17.1 ± 12.7*	15.9 ± 10.5^†^	0.695

Data are presented as the mean ± SD or number (percentage). nOH, neurogenic orthostatic hypotension; SBP, systolic blood pressure; DBP, diastolic blood pressure; HR, heart rate; OHQ, OH questionnaire; OHDAS, orthostatic hypotension daily activity scale; OHSA, orthostatic hypotension symptom assessment, BDI, Beck depression inventory.

a *P* value from Mann–Whitney *U* test or chi‐square test.

*P < 0.05, ^†^ *P* < 0.01 compared with the baseline, Wilcoxon signed‐rank test.

Repeated‐measures ANOVA revealed significant time effects on orthostatic SBP drops, DBP drops, orthostatic HR changes and supine HR. No significant effect was found according to the treatment group or group by time interaction (Table S1).

### Atomoxetine, but not midodrine, improved OH‐related symptoms at the 1‐month follow‐up visit

Only the atomoxetine group showed improvement in the total OHQ and in both OHDAS and OHSA component score compared with the baseline. The BDI‐II improved in both treatment groups compared to the baseline. Repeated‐measures ANOVA revealed significant time effects on all questionnaire scores (Fig. 2, Table 2). No significant effect by group or group by time interaction was found (Table S1).

### Number of patients with OH at the 1‐ and 3‐month follow‐up visits

At 1 month after treatment, 42 patients were evaluated, and 11 (26.2%) met the criteria for OH. The proportion of the patients who met BP criteria did not differ among treatment groups at 1 month (*P* = 0.47) (Table 2). They received both atomoxetine and midodrine, and 10 were followed for an additional 2 months. Except for one patient who initially received midodrine, all still met the criteria for OH at 3 months. Among those who did not have OH at 1 month, three of those who received atomoxetine and one who received midodrine had OH at 3 months (Fig. 1).

A total of 15 patients were lost to follow‐up at 3 months. They had higher supine SBP values at the baseline compared with those who completed the study (140.8 ± 20.1 vs. 126.1 ± 17.4, *P* = 0.026). There were no significant differences in demographics, initial orthostatic vital signs, questionnaire scores, or treatment modalities (Table S2).

### OH‐related symptoms gradually improved over the course of 3 months in both the atomoxetine and midodrine groups

Patients who continued atomoxetine single treatment had further improvement in their total OHQ score (29.9 ± 18.4 vs. 21.1 ± 17.5, *P* = 0.02) and OH‐related symptom severity (OHSA: 18.6 ± 12.7 vs. 14.2 ± 9.1, *P* = 0.014) compared to that measured at 1 month. Those who continued midodrine monotherapy showed further improvement in their total OHQ score (36.4 ± 24.8 vs. 16.3 ± 19.0, *P* = 0.003) and in OH‐related restriction in daily activity (OHDAS: 14.9 ± 12.1 vs. 8.3 ± 8.6, *P* = 0.007) and had a trend toward improvement in their OH‐related symptoms severity (OHSA: 21.6 ± 13.6 vs. 14.2 ± 11.6, *P* = 0.054) at 3 months. No significant changes in orthostatic vital signs and questionnaire score were observed in those who received the combination treatment (Table 3).

**Table 3:** Orthostatic vital signs and Questionnaire scores at 1 and 3 months.

	Continued atomoxetine after 1 month	Continued midodrine after 1 month	Received combination after 1 month
No. of patients with nOH			
1 month	0/13	0/18	11/11
3 month	3/12	1/13	9/10
Orthostatic SBP drop (mmHg)			
1 month	6.3 ± 5.2	9.2 ± 6.9	32.6 ± 17.3
3 month	8.1 ± 10.6	8.9 ± 7.6	26.9 ± 15.2
Orthostatic DBP drop (mmHg)			
1 month	−0.3 ± 4.9	−1.2 ± 4.8	14.5 ± 8.9
3 month	2.7 ± 5.6	1.2 ± 5.8	12.8 ± 9.8
Orthostatic HR increase			
1 month	5.9 ± 5.7	7.0 ± 4.9	10.6 ± 10.4
3 month	3.8 ± 3.3	5.6 ± 7.9	7.4 ± 6.2
Supine SBP (mmHg)			
1 month	131.0 ± 15.5	132.3 ± 19.1	126.6 ± 22.9
3 month	138.9 ± 16.9	125.9 ± 17.6	125.0 ± 26.7
Supine DBP (mmHg)			
1 month	82.2 ± 12.1	80.7 ± 11.5	76.8 ± 11.3
3 month	85.8 ± 12.9	80.0 ± 14.9	77.0 ± 15.8
Supine HR			
1 month	81.2 ± 16.5	75.0 ± 9.8	76.8 ± 14.6
3 month	86.2 ± 11.2	75.3 ± 12.8	82.1 ± 7.7
OHQ total			
1 month	29.9 ± 18.4	36.4 ± 24.8	35.2 ± 21.1
3 month	21.1 ± 17.5*	16.3 ± 19.0^†^	34.1 ± 29.5
OHDAS			
1 month	11.2 ± 7.7	14.9 ± 12.1	16.3 ± 7.6
3 month	8.7 ± 9.8	8.3 ± 8.6^†^	14.2 ± 13.4
OHSA			
1 month	18.6 ± 12.7	21.6 ± 13.6	18.9 ± 14.4
3 month	14.2 ± 9.1*	14.2 ± 11.6	23.3 ± 16.8
BDI			
1 month	12.8 ± 7.8	16.4 ± 11.7	20.8 ± 13.8
3 month	10.8 ± 7.8	15.0 ± 10.4	17.0 ± 13.7

Data are presented as the mean ± SD or number (percentage). nOH, neurogenic orthostatic hypotension; SBP, systolic blood pressure; DBP, diastolic blood pressure; HR, heart rate; OHQ, OH questionnaire; OHDAS, orthostatic hypotension daily activity scale; OHSA, orthostatic hypotension symptom assessment, BDI, Beck depression inventory.

*P < 0.05, ^†^ *P* < 0.01 compared with the 1 month, Wilcoxon signed‐rank test.

### Adverse events

Five (5/42, 11.9%) of the patients who were reevaluated at 1 month reported adverse events. Two of them received midodrine (2/19, 10.5%), and three received atomoxetine (3/23, 13.0%). Of those who received midodrine, one complained of worsening dizziness, and the other experienced agitation. Of the three who received atomoxetine, two had frequent sweating, and one had frequent urination. They all dropped out after 1 month of treatment. No additional adverse events were reported at the 3‐month follow‐up visit.

Proportion of patients with supine hypertension was similar between the treatment groups at 1‐ and 3‐month follow‐up (Table S3).

## Discussion

Atomoxetine therapy resulted in a similar improvement in orthostatic BP changes compared to those induced by midodrine after 1 month of use. Only 31.6% of those who received atomoxetine met the criteria for OH at 1 month, which was statistically comparable to the proportion of patients taking midodrine who still had OH after 1 month of treatment (21.7%). Atomoxetine improved OH‐related symptoms at 1 month, while midodrine resulted in no significant changes. The extended use of atomoxetine or midodrine for 3 months in those who showed a response at 1 month showed additional improvements in OH‐related symptoms. The combination of midodrine and atomoxetine in monotherapy‐resistant OH patients demonstrated no additional benefit with regard to improving orthostatic BP drops and OH‐related symptoms. This study is the first to demonstrate the efficacy and safety of atomoxetine in patients with nOH after treatment for up to 3 months.

Atomoxetine improved the standing SBP and DBP drop after 1 month of treatment, which was comparable to that achieved with midodrine therapy. An acute increase in upright SBP after atomoxetine treatment was previously reported in several studies.^11^, ^12^, ^13^ However, there has been limited evidence regarding the effects of the long‐term use of atomoxetine on OH. Only one case report of an 84‐year‐old man showed a beneficial effect of atomoxetine therapy for up to 10 weeks.^18^ Our results show that atomoxetine can ameliorate orthostatic SBP and DBP drops for up to 3 months without any significant adverse events and without affecting supine BP.

The improvements in the OHQ score, which evaluates OH‐related symptom severity and restriction in daily activity, were significant only in the atomoxetine group at 1 month. This result is in line with the finding in a previous study that suggesting that atomoxetine was better at ameliorating orthostatic symptoms than midodrine.^11^ Atomoxetine is a psychostimulant that can increase right inferior frontal activation.^19^ Orthostatic intolerance may result from a decrease in cerebral blood flow while standing, and atomoxetine can improve the blood flow not only by increasing systemic BP but also by directly modulating the cerebral blood flow.^20^ Moreover, atomoxetine is classified as a selective noradrenaline reuptake inhibitor and has anti‐depressive properties.^21^ Compared to our previous study, the midodrine group showed less improvement in OH‐related symptoms at 1 month. Patients who received midodrine in this study had more severe depression than those in a previous study (BDI‐II score 21.3 ± 15.1 vs. 13.6 ± 6.8) and more had central autonomic dysfunction (12% vs. 3.4%), which could have resulted in the discrepancy.

The extended use of atomoxetine for up to 3 months resulted in additional improvement in the OHSA score in those who showed a response at 1 month. Our results support the finding in a previous case report of an elderly patient with OH that atomoxetine can gradually improve orthostatic BP and its symptoms over a 10‐week period.^18^ Meanwhile, three of the patients still fulfilled the criteria for OH after 3 months, despite an overall improvement in orthostatic BP drop and the related symptoms. Atomoxetine can cause central inhibition of the sympathetic nervous system, which can counteract the pressor effect of the drug.^22^ Long‐term use of atomoxetine may have ameliorated its pressor effect in selected patients. Consistent improvement in OH‐related symptoms with midodrine treatment for up to 3 months is in line with the results of our previous study,^8^ which emphasizes the necessity for the long‐term use of midodrine for at least 3 months.

The combination of atomoxetine and midodrine resulted in no additional benefit in those who did not show an initial response to a monotherapy. Atomoxetine has a synergistic effect with medications that can enhance norepinephrine release in neurovascular junctions in different mechanisms, such as pyridostigmine^23^ or yohimbine.^13^ Midodrine, however, works directly on the adrenergic receptor at vascular walls and increases vessel tone. Because atomoxetine and midodrine both activate postganglionic sympathetic neurons, combination treatment may have limited additional benefit. Further study comparing the efficacy between single and combination treatments with atomoxetine and midodrine are warranted.

It has been suggested that preserved peripheral noradrenergic autonomic nerve function is essential for atomoxetine to show its efficacy. Only patients with central autonomic failure, but not those with peripheral autonomic failure, exhibited improvement in standing SBP at 1 h after the administration of atomoxetine.^12^ In our study, more than half of the patients lacked a definite etiology of OH and included those with diabetic and nondiabetic peripheral neuropathy. We did not perform a subgroup analysis according to etiology because only 16% of our patients had definite central autonomic failure. Although this study was a single‐center study without a placebo group and a blinding process, it was the first to evaluate the long‐term efficacy of atomoxetine for the treatment of OH. Additional studies evaluating the long‐term efficacy of atomoxetine in patients with homogenous OH etiology may be warranted.

Atomoxetine may be a better alternative to midodrine for the management of nOH in the short term. One month of treatment with atomoxetine improved orthostatic BP drop similar to 1 month of treatment with midodrine without severe adverse events, and the OH‐related symptoms improved significantly only in the atomoxetine group. Treatment response at 1 month was an important marker for making clinical decisions regarding OH management. For those who showed a response to atomoxetine at 1 month, it was safe and beneficial to maintain the treatment for up to 3 months. Further larger randomized double‐blinded placebo‐controlled studies with longer follow‐up periods are necessary to confirm the short‐ and long‐term efficacies of atomoxetine and midodrine.

## Conflicts of Interest

The authors declare that they have no competing interests.

## Supporting information


**Table S1.** Result of repeated‐measures ANOVA.
**Table S2.** Characteristics of patients who dropped out.
**Table S3.** Proportion of patients with supine hypertension.
