# Supplementary material for: Efficacy of atomoxetine versus midodrine for neurogenic orthostatic hypotension
Source: Ann Clin Transl Neurol. 2019 Dec 19;7(1):112–20. doi: 10.1002/acn3.50968 (PMC6952305; doi:10.1002/acn3.50968)
Supplement: Supplementary file 1 — Table S1. Result of repeated‐measures ANOVA. Table S2. Characteristics of patients who dropped out. Table S3. Proportion of patients with supine hypertension. [file ACN3-7-112-s001.doc]

**Supplementary Table 1. Result of repeated-measures ANOVA**

|  | Group | Time | Time x Group |
| --- | --- | --- | --- |
| Supine SBP (mmHg) | ns | ns | ns |
| Supine DBP (mmHg) | ns | ns | ns |
| Supine HR | ns | F (1,40)=20.485, p<0.001 | ns |
| Orthostatic SBP change (mmHg) | ns | F (1,40)=32.3, p<0.001 | ns |
| Orthostatic DBP change (mmHg) | ns | F (1,40)=56.341, p<0.001 | ns |
| Orthostatic HR change | ns | F (1,40)=8.037, p=0.007 | ns |
| OHQ total | ns | F (1,40)=9.546, p=0.004 | ns |
| OHDAS | ns | F (1,40)=4.551, p=0.039 | ns |
| OHSA | ns | F (1,40)=8.650, p=0.005 | ns |
| BDI | ns | F (1,40)=18.231, p <0.001 | ns |

Group: Atomoxetine and Midodrine; Time: Baseline, 1 month.

Abbreviations: SBP, systolic blood pressure; DBP, diastolic blood pressure; OHDAS, orthostatic hypotension daily activity scale; OHSA, orthostatic hypotension symptom assessment; SF-36v2, Short Form (36) Health Survey version 2

**Supplementary Table 2.** Characteristics of patients who dropped out

|  | Completed | Dropout | p-value |
| --- | --- | --- | --- |
|  | 35 | 15 |  |
| Age (years) | 61.6±10.5 | 66.7±5.7 | 0.075 |
| Sex (male) | 19 (54.3) | 9 (60.0) | 0.709 |
| BMI (kg/m2) | 23.6±5.1 | 25.5±3.5 | 0.295 |
| Etiology |  |  | 0.136 |
| -Idiopathic OH | 17 (48.6) | 12 (80.0) |  |
| -MSA | 6 (17.1) | 2 (13.0) |  |
| -Diabetic AN | 8 (22.9) | 0 |  |
| -Nondiabetic AN | 4 (11.4) | 1 (6.7) |  |
| Initial vital signs |  |  |  |
| **Supine SBP (mmHg)** | **126.1±17.4** | **140.8±20.1** | **0.026** |
| Supine DBP (mmHg) | 78.5±10.6 | 84.3±10.1 | 0.09 |
| Supine HR | 68.7±14.2 | 67.1±10.8 | 0.857 |
| Ortho SBP drop (mmHg) | 26.1±11.6 | 26.2±6.6 | 0.581 |
| Ortho DBP drop (mmHg) | 13.4±8.6 | 10.9±8.9 | 0.703 |
| Ortho HR change | 12.0±10.2 | 11.6±6.5 | 0.362 |
| Initial Questionnaires |  |  |  |
| OHDAS | 16.2±10.7 | 14.7±11.1 | 0.61 |
| OHSA | 23.1±13.9 | 22.9±13.9 | 0.957 |
| BDI | 20.4±13.4 | 17.3±7.7 | 0.696 |
| Treatments |  |  | 0.758 |
| Atomoxetine | 17 (48.6) | 8 (53.3) |  |
| Midodrine | 18 (51.4) | 7 (46.7) |  |

Data are presented as the mean±SD or number (percentage).

Abbreviations: BMI, body weight index, OH, orthostatic hypotension; MSA, multiple system atrophy; AN, autonomic neuropathy, SBP, systolic blood pressure; DBP, diastolic blood pressure; HR,heart rate; OHDAS, orthostatic hypotension daily activity scale; OHSA, orthostatic hypotension symptom assessment, BDI, Beck depression inventory

**Supplementary Table 3. Proportion of patients with supine hypertension.**

|  | Total | Atomoxetine | Midodrine | p-value1) |
| --- | --- | --- | --- | --- |
| Baseline | 10/50 (20.0) | 4/25 (16.0) | 6/25 (24.0) | 0.703 |
| 1 month | 8/42 (19.0) | 3/19 (15.8) | 5/23 (21.7) | 0.625 |
|  |  |  |  |  |
|  | Continued  Atomoxetine  after 1 month | Continued  Midodrine  after 1 month | Received  Combination  after 1 month |  |
| 1 month | 3/13 (23.1) | 4/18 (22.2) | 1/11 (9.1) | 0.618 |
| 3 month | 5/12 (41.7) | 4/13 (30.8) | 2/10 (20.0) | 0.551 |

Supine hypertension defined by supine SBP>150mmHg or supine DBP>90mmHg

Data are presented as number (percentage).

^1)^ p-value from Chi-square test
